# Dysregulation of miR-138-5p/RPS6KA1-AP2M1 Is Associated With Poor Prognosis in AML

**DOI:** 10.3389/fcell.2021.641629

**Published:** 2021-02-26

**Authors:** Dong-Hu Yu, Chen Chen, Xiao-Ping Liu, Jie Yao, Sheng Li, Xiao-Lan Ruan

**Affiliations:** ^1^Department of Biological Repositories, Human Genetics Resource Preservation Center of Hubei Province, Zhongnan Hospital of Wuhan University, Wuhan, China; ^2^The Second Clinical College, Wuhan University, Wuhan, China; ^3^Department of Urology, Zhongnan Hospital of Wuhan University, Wuhan, China; ^4^Department of Hematology, Renmin Hospital of Wuhan University, Wuhan, China

**Keywords:** acute myeloid leukemia, GSVA, PI3K-Akt-mTOR pathway, prognosis, RPS6KA1, AP2M1, WGCNA, chemoresistance

## Abstract

Acute myeloid leukemia (AML) is a malignant disease of hematopoietic stem/progenitor cells, and most AML patients are in a severe state. Internal tandem duplication mutations in FLT3 gene (FLT3-ITD) detected in AML stem cells account for 20–30 percent of AML patients. In this study, we attempted to study the impact of the interaction of FLT3-ITD mutation and the CXCL12/CXCR4 axis in AML, and the possible mechanisms caused by the impact by bioinformatics. Gene set variation analysis (GSVA) revealed that the PI3K-Akt-mTOR pathway positively correlated with the status of FLT3-ITD mutation. Multiple survival analyses were performed on TCGA-AML to screen the prognostic-related genes, and RPS6KA1 and AP2M1 are powerful prognostic candidates for overall survival in AML. WGCNA, KEGG/GO analysis, and the functional roles of RPS6KA1 and AP2M1 in AML were clarified by correlation analysis. We found that the expression levels of RPS6KA1 and AP2M1 were significantly associated with chemoresistance of AML, and the CXCL12/CXCR4 axis would regulate RPS6KA1/AP2M1 expression. Besides, miR-138-5p, regulated by the CXCL12/CXCR4 axis, was the common miRNA target of RPS6KA1 and AP2M1. Taken together, the interaction of FLT3-ITD mutation and the CXCL12/CXCR4 axis activated the PI3K-Akt-mTOR pathway, and the increased expression of RPS6KA1 and AP2M1 caused by hsa-miR-138-5p downregulation regulates the multi-resistance gene expression leading to drug indications.

## Introduction

Acute myeloid leukemia (AML) is a kind of hematopoietic system malignant hyperplasia disease (Cai and Levine, 2019), and the different genetic backgrounds of AML patients lead to various prognoses. The commonest genetic alteration is internal tandem duplication mutation in the FLT3 gene (FLT3-ITD), and FLT3-ITD is found in about 20–30 percent AML patients. AML patients with FLT3-ITD mutation have a higher relapse rate, more resistance to chemotherapy, and poorer survival than AML patients without FLT3-ITD mutation (Eldfors et al., 2014; Garg et al., 2015). Chemokine ligand 12 (CXCL12), also named stromal-derived factor 1 (SDF-1α), can induce tumor cell migration in AML, and chemokine receptor 4 (CXCR4) is the specific receptor for CXCL12 (Chatterjee et al., 2014). The signal pathways activated by the CXCL12/CXCR4 axis have been demonstrated to show effects in interactions between tumor cells and the microenvironment in AML, and the interactions would cause chemoresistance and AML relapse (Lee et al., 2017; Cao et al., 2019b). Several recent publications suggest an interaction between FLT3-ITD mutation and CXCL12/CXCR4 axis, and the CXCL12/CXCR4 axis has been proved an important role in the rapid emergence of resistance to FLT3 inhibitors (Cao et al., 2019b; Kim et al., 2020; Waldeck et al., 2020). Although it has been identified that the combination of the FLT3 inhibitor and CXCR4 antagonist could improve the sensitivity of chemotherapy treatment in AML cells (Jacobi et al., 2010; Kim et al., 2020), the lack of understanding of the impact on AML cells produced by the interaction between FLT3-ITD mutation and the CXCL12/CXCR4 axis restricts the development of the related research. Therefore, it is important to identify the downstream signaling events as a result of the interaction.

The PI3K–AKT–mTOR pathway is one of the intracellular signaling pathways that have been identified as important in cancer (Herschbein and Liesveld, 2018; Liu et al., 2019). Recent studies show that the PI3K–AKT–mTOR pathway plays a pivotal part in chemoresistance and the self-renewal of tumor stem cells, which is considered as the major cause of cancer metastasis, recurrence, and treatment failure (Simioni et al., 2019; Evangelisti et al., 2020). The CXCL12/CXCR4 axis could effectively activate the PI3K–Akt–mTOR pathway in many types of cancers, such as lung cancer, gastric cancer, and glioma (Chen et al., 2012; Luo et al., 2013; Zhan et al., 2016). Besides, the PI3K–Akt–mTOR pathway is important for the proliferation, survival, and migration of FLT3-ITD AML cells (Chen et al., 2010). Therefore, the PI3K–Akt–mTOR pathway, as an essential downstream pathway in AML, should be further explored to know more about the impact of the interaction between FLT3-ITD mutation and the CXCL12/CXCR4 axis in AML.

In the present study, we found that the PI3K–AKT–mTOR pathway was activated most significantly in AML patients with FLT3-ITD mutation by using multiple datasets from Gene Expression Omnibus (GEO). Survival analyses were performed for the key genes in the PI3K–AKT–mTOR pathway, and we found that high expression levels of RPS6KA1 and AP2M1 suggested poor prognosis in AML patients. The expression levels of RPS6KA1 and AP2M1 were significantly correlated with the expression levels of some drug-resistance-associated genes, and the targeted interventions of RPS6KA1 and AP2M1 might improve the chemosensitivity in AML cells. Besides, the common upstream miRNA (miR-138-5p) of RPS6KA1 and AP2M1 was predicted by microT-CDS and TargetScan. The established miR-138-5p/RPS6KA1-AP2M1 pathway would provide more effective therapeutic targets and some useful clues for the molecular mechanism of AML chemoresistance.

## Materials and Methods

### Data Collection

**FIGURE 1:**
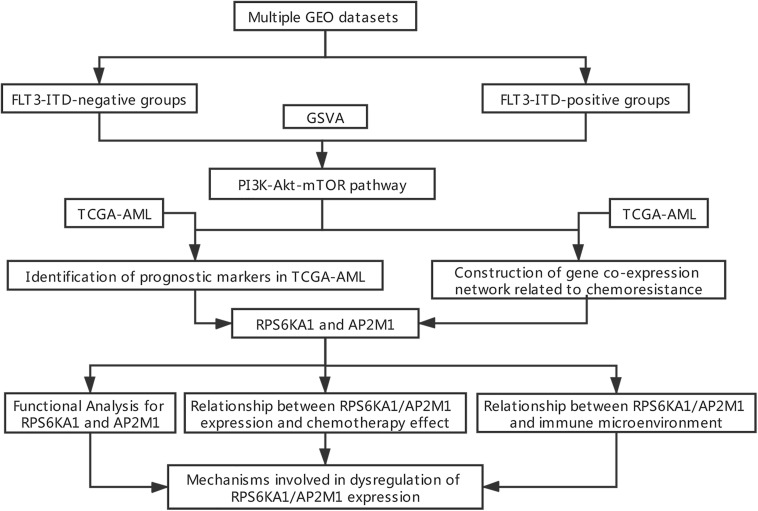
Flowchart of data processing and analysis.

A brief workflow for this study is shown in Figure 1. The normalized TCGA-AML dataset was downloaded from UCSC Xena^1^, and other datasets (GSE6891, GSE10358, GSE15434, GSE61804, GSE64623, GSE76004, GSE76008, GSE106291, and GSE44842) were downloaded from the GEO database^2^. The details of these GEO datasets are listed in Supplementary Table 1.

### Gene Set Variation Analysis (GSVA)

The samples were divided from the GSE6891, GSE10358, GSE15434, and GSE61804 datasets into FLT3-ITD-negative groups and FLT3-ITD-positive groups, separately. GSVA was conducted between FLT3-ITD-negative groups and FLT3-ITD-positive groups in R (Haenzelmann et al., 2013). The hallmark gene sets in MSigDB^3^ were picked as the reference gene sets, and *t*-value > 2 was the threshold. The common activated and suppressed signaling pathways in four GEO datasets were selected.

### Survival Analyses for AML Patients

In our study, “HALLMARK_ PI3K_ AKT_ MTOR SIGNALING” was significantly activated in FLT3-ITD-positive AML patients, and the genes in this pathway were selected as the candidate genes for further research. Univariate Cox regression analysis was performed for the candidate genes to screen the genes associated with OS in TCGA-AML. LASSO regression analysis was performed for the candidate genes by “glmnet” package in R to further screen the genes related to OS (Peinemann and Labeit, 2018; Engebretsen and Bohlin, 2019). Then, multivariate Cox regression analysis was carried out to identify the hub genes as the independent prognosis factors in AML. Moreover, the combined effects of RPS6KA1 and AP2M1 expression levels and FLT3-ITD mutation on survival were demonstrated in UALCAN. The UALCAN database^4^ is a convenient portal for analyzing the relationships between gene expression levels and clinical features in various cancers, and we can have easy access to the information of cancer transcriptome data and clinical data including AML.

### Weighted Gene Co-expression Network Analysis (WGCNA)

Weighted gene co-expression network analysis (WGCNA) is a widely used data mining method based on the correlation coefficient between two variables, and it can be used to analyze the relationships between clinical characteristics and gene expression levels in this study (Langfelder and Horvath, 2008). According to the results of variance analysis of GSE106291, the top 25% most variant genes were conducted into a gene co-expression network. There are six main steps for WGCNA in R: (1) constructing a similarity matrix with the correlations of the genes, (2) selecting an appropriate power of β as the soft thresholding parameter, (3) constructing an adjacency matrix, (4) transforming the adjacency matrix into a topological overlap matrix (TOM), (5) identifying the relevant dissimilarity (1-TOM), and (6) categorizing the genes with similar expression levels into the same module. The module including RPS6KA1 and AP2M1 was selected for further analyses.

### Drug Sensitivity Analysis

Based on the ridge regression model in Genomics of Drug Sensitivity in Cancer (GDSC)^5^, the half-inhibitory concentrations (IC50) of some certain drugs for the patients in TCGA-AML were estimated by “pRRophetic” package in R (Geeleher et al., 2014). The differences in IC50 of certain drugs between the RPS6KA1/AP2M1 high and low expression groups were compared by Student’s *t*-tests. To further explore the possible mechanisms of different IC50 in the certain drugs, the correlations between RPS6KA1/AP2M1 and well-known drug-resistance-associated genes in AML were calculated. In addition, gene network analysis was performed to explore the correlations between RPS6KA1/AP2M1 and the chemoresistance molecules, such as transcription factors and in GenCLiP 3.0^6^. GenCLip 3.0, a newly online tool, offers the analysis of the gene network to help the users understand the gene regulatory mechanism.

### Gene Signature Analysis

To explore the function of RPS6KA1 and AP2M1 in AML chemoresistance, pairwise gene expression level correlation analysis of RPS6KA1 and AP2M1 was applied in chemotherapy-resistant patients of GSE106291 by Spearman’s correlation analysis. According to the absolute values of correlation coefficients, we selected the top 500 genes to conduct functional enrichment analysis. Then, we could estimate the potential functions of RPS6KA1 and AP2M1; this type of analysis was described as “guilt of association.”

### Gene Expression Profiling Interactive Analysis (GEPIA) Database Analysis

The GEPIA database^7^ is a newly developed online server providing the analyses of the comprehensive RNA sequencing expression data [including 8,587 normal samples from Genotype-Tissue Expression (GTEx) and 9736 tumors from TCGA], and it contains gene expression correlation analysis, similar gene expression detection, and so on. The associations between the important marker of leukemia stem cells (KAT7) and RPS6KA1/AP2M1 and between CXCR4 and RPS6KA1/AP2M1 were calculated in GEPIA.

### Immune Microenvironment Landscape Exploration

To further explore the immune microenvironment in AML, the stromal score, the immune score, and the tumor purity of each patient in TCGA-AML were evaluated by the “estimate” package in R. Besides, the relative abundances of 22 immune cells in each patient were computed by “Cell type Identification by Estimating Relative Subsets of RNA Transcripts (CIBERSORT)”^8^. The differences of immune microenvironment between the RPS6KA1/AP2M1 high and low expression groups were compared by Student’s *t*-tests. Moreover, single-sample GSEA (ssGSEA) was performed to calculate the relative abundances of 24 immune cells for TCGA-AML, and the correlations between the abundance of tumor-infiltrating immune cells and the expression levels of RPS6KA1 and AP2M1 were identified by Spearman’s correlation analysis.

### Cancer Single-Cell State Atlas (CancerSEA) Database Analysis

CancerSEA^9^ is a dedicated database aiming to explore the different functional states (including 14 cell functional states) of cancer cells at the single-cell level. CancerSEA contains more than 40,000 cancer single cells in different cancers, and the correlations between gene expression levels and functional states would be provided in this database. We identified the functional states of RPS6KA1 and AP2M1 in AML by using CancerSEA.

### miRNA Prediction

Two web tools, TargetScan^10^ and microT-CDS^11^, were used to predict the miRNAs for RPS6KA1 and AP2M1. Selection for miRNA criteria was as follows: (1) the score of microT-CDS > 0.7; (2) the context++ score percentile in TargetScan > 85. The common miRNAs of RPS6KA1 and AP2M1 were defined as the key miRNAs for further study.

### Differential Expression Analysis

The R package “limma” was used to identify differentially expressed miRNAs between control group and the treatment group in GSE44828 (P.val < 0.05; log_2_| FC| > 1). The OCI-AML3 cells in treatment groups in GSE44828 were given 250 nm POL6326 (a type of CXCR4 antagonists) or 100 ng/ml SDF-1 α (the recombinant human CXCL12). Moreover, we intersected the miRNAs with SDF-1 α downregulation and POL6326 upregulation to select the miRNAs regulated by the CXCL12/CXCR4 axis.

## Results

### The FLT3-ITD Mutation-Activated PI3K-AKT-mTOR Pathway

**FIGURE 2:**
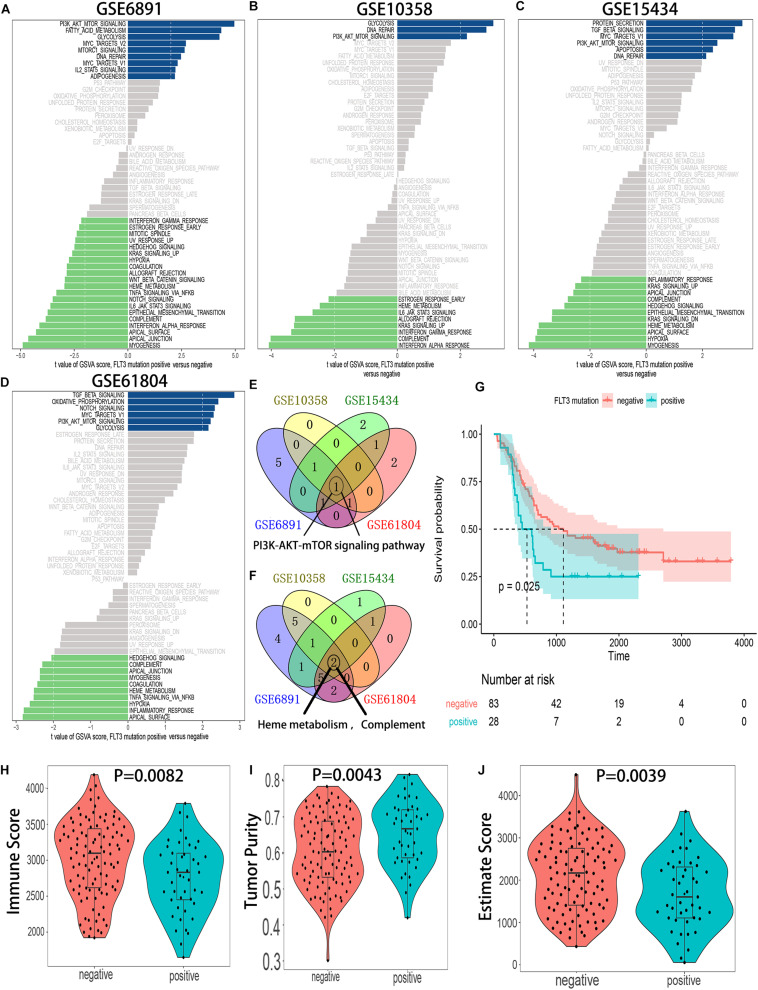
The PI3K–AKT–mTOR pathway is significantly activated in the AML patients with FLT3-ITD mutation. **(A)** GSVA of the GSE6891 dataset. **(B)** GSVA of the GSE10358 dataset. **(C)** GSVA of the GSE15434 dataset. **(D)** GSVA of the GSE61804 dataset. **(E)** Venn diagram of the activated gene sets in the indicated datasets. **(F)** Venn diagram of the suppressed gene sets in the indicated datasets. **(G)** KM survival analysis of the FLT3-ITD-positive group and FLT3-ITD-positive group in GSE76004. **(H)** Differences in immune score between AML patients with FLT3-ITD-positive and FLT3-ITD-negative. **(I)** Differences in tumor purity between AML patients with FLT3-ITD-positive and FLT3-ITD-negative. **(J)** Differences in estimate score between AML patients with FLT3-ITD-positive and FLT3-ITD-negative.

Gene set variation analysis of hallmark gene sets was conducted in 4 independent GEO datasets: GSE6891, GSE10358, GSE15434, and GSE61804. The results showed that the PI3K-AKT-mTOR pathway was significantly activated in FLT3-ITD-positive AML patients (Figures 2A–F), and the detailed results of GSVA for 4 datasets are shown in Supplementary Tables 2–5. Compared with the OS, tumor purity, and immune score in the FLT3-ITD-negative group, the patients in the FLT3-ITD-positive group had lower OS, lower immune score, and higher tumor purity (Figures 2G–J). Based on the results of GSVA, survival analysis, and immune microenvironment analysis, we speculated that the genes in the PI3K–AKT–mTOR signaling pathway might be effective prognostic candidates, and they might have an important impact on the immune microenvironment in AML.

### Identification of the Genes Related to Prognosis in TCGA-AML

**FIGURE 3:**
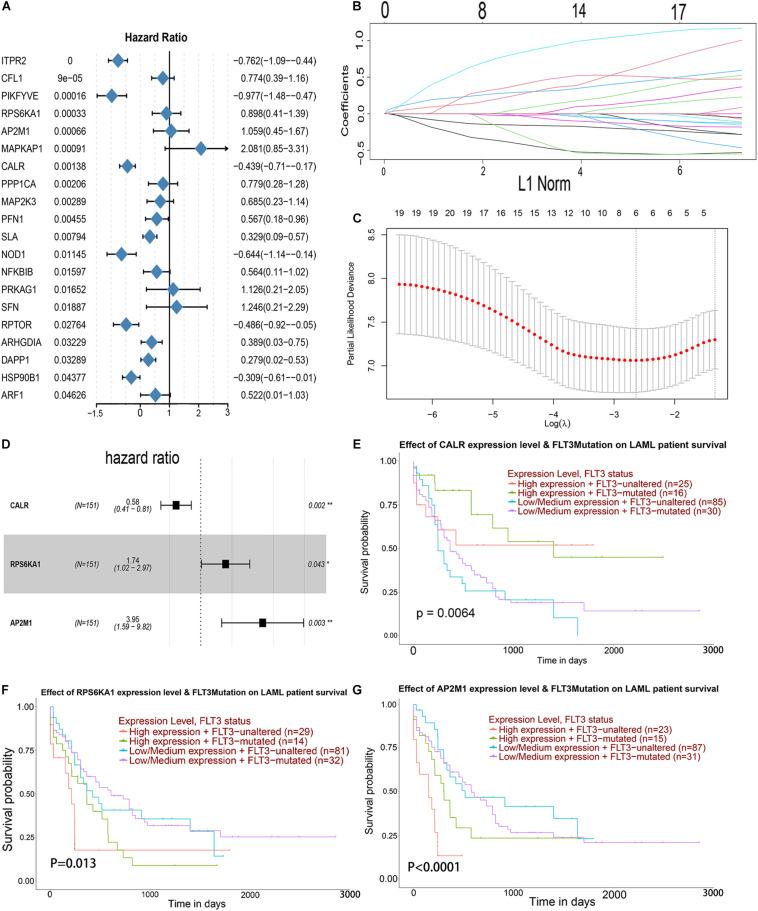
Identification of prognostic markers in TCGA-AML. **(A)** Forest map of the genes related to AML survival, analyzed by univariate Cox regression. **(B)** A coefficient distribution map for a logarithmic (λ) sequence by LASSO. **(C)** Selecting the best parameters for AML in the LASSO model (λ). **(D)** Forest map of the genes related to AML survival, analyzed by multivariate Cox regression. **(E)** The comprehensive effects of the mutational status of FLT3 and the expression levels of CALR in the UALCAN database. **(F)** The comprehensive effects of mutational status of FLT3 and the expression levels of RPS6KA1 in the UALCAN database. **(G)** The comprehensive effects of mutational status of FLT3 and the expression levels of AP2M1 in the UALCAN database.

Univariate Cox analysis was performed for 105 genes in the PI3K–AKT–mTOR pathway from the MSigdb database^12^, and 20 genes were associated with OS in AML patients (Figure 3A). LASSO regression analysis was conducted for these 20 genes, and 6 genes was selected (Figures 3B,C). Then, multivariate Cox regression analysis was applied for 6 genes, and 3 genes (CALR, RPS6KA1, and AP2M1) related to the prognosis of AML were obtained as hub genes (Figure 3D). Regardless of whether FLT3 is mutated or not, the patients with low expression level of CALR, high expression level of RS6KA1, or high expression level of AP2M1 had a lower survival rate. Low expression level of CALR, high expression levels of RPS6KA1 and RPS6KA1, and FLT3 mutations prompt worse prognosis (Figures 3E–G).

### Identification of the Module-Related Chemotherapy Resistance in AML by WGCNA

**FIGURE 4:**
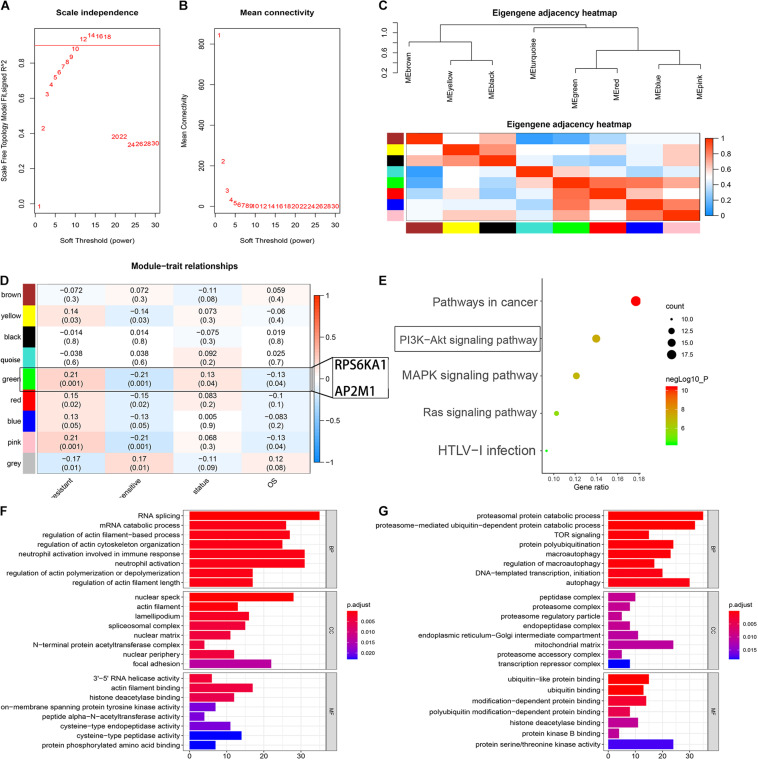
Identification of the module-related chemotherapy resistance in AML by WGCNA. **(A)** Analysis of the scale-free fit index for various soft-thresholding powers. **(B)** Analysis of the mean connectivity for various soft-thresholding powers. **(C)** Heat map of the eigengene adjacency. **(D)** Heat map of the correlation between co-expressed gene module eigengenes and clinical traits of AML. **(E)** KEGG pathway enrichment of the genes in the green module. **(F)** Function analysis for the gene RPS6KA1 by guilt of association. **(G)** Function analysis for the gene AP2M1 by guilt of association.

To identify the modules associated with the chemotherapy sensitivity and prognosis in AML, the gene expression profile and relative clinical information (including the responses to chemotherapy, survival status, and OS) in GSE106291 were collected for WGCNA. We selected β = 12 (scale free *R*^2^ = 0.92) as the soft-thresholding parameter to construct the gene co-expression network (Figures 4A,B). As a result, 8 modules were recognized after dynamic tree cut merging (Figure 4C), and the correlations between each module and the status of chemotherapy resistance, between each module and survival status, and between each module and OS for the patients were evaluated. The green module was identified as the hub module significantly correlated with the responses to chemotherapy, OS time, and OS status (*P* < 0.05) (Figure 4D). Based on DAVID^13^, GO and KEGG enrichment analyses were performed to clarify the biological meaning of the genes in the green module, and the top five significantly enriched pathways were pathways in cancer, PI3K-Akt signaling pathway, MAPK signaling pathway, Ras signaling pathway, and HTLV-I infection (Figure 4E). The terms of BP for the genes in the green module mainly contained peptidyl-serine phosphorylation, G1/S transition of mitotic cell cycle, and positive regulation of cell proliferation; CC terms for the genes in the green module were extracellular exosome, cytosol, nucleolus, and so on; MF terms were mainly enriched in ATP binding, protein serine/threonine kinase activity, and cyclin-dependent protein serine/threonine kinase activity (Supplementary Tables 6–8).

### Functional Analysis for RPS6KA1 and AP2M1

According to the results of “guilt of association” analysis for GSE106291, RPS6KA1 was essential for RNA splicing, mRNA catabolism, and regulation of actin filament-based processes; AP2M1 mainly played important roles in proteasomal protein catabolic processes, proteasome-mediated ubiquitin-dependent protein catabolic processes, and TOR signaling (Figures 4F,G).

### RPS6KA1/AP2M1 Expression Levels Are Correlated With Clinical Features of AML

Based on the median expression levels of RPS6KA1 and AP2M1, the patients of TCGA-AML were divided into two groups separately. According to the results of Chi square tests, we demonstrated that a higher expression of RPS6KA1 was correlated with poorer OS (*P* = 0.013) and poorer survival status (*P* = 0.047), and the expression level of RPS6KA1 increased in the AML patients with M5 (Table 1). A higher expression of AP2M1 was correlated with poorer OS (*P* < 0.0001) and poorer survival status (*P* = 0.047). The expression level of AP2M1 reduced in the AML patients with M0, while it increased in the patients with M5 (Table 2).

**TABLE 1:** RPS6KA1 expression is associated with multiple clinical and molecular characteristics of AML.

		RPS6KA1 expression level		
		
		High (*n* = 67)	Low (*n* = 67)	*P*
Gender (%)	FEMALE	31 (46.3)	30 (44.8)	1
	MALE	36 (53.7)	37 (55.2)	
Status (%)	Alive	18 (26.9)	30 (44.8)	0.047
	Dead	49 (73.1)	37 (55.2)	
OS [mean (*SD*)]		431.54 (484.52)	681.24 (651.19)	0.013
M0 (%)	M0	7 (10.4)	7 (10.4)	1
	Non-M0	60 (89.6)	60 (89.6)	
M1 (%)	M1	15 (22.4)	17 (25.4)	0.839
	Non-M1	52 (77.6)	50 (74.6)	
M2 (%)	M2	11 (16.4)	19 (28.4)	0.147
	Non-M2	56 (83.6)	48 (71.6)	
M3 (%)	M3	7 (10.4)	7 (10.4)	1
	Non-M3	60 (89.6)	60 (89.6)	
M4 (%)	M4	15 (22.4)	13 (19.4)	0.832
	Non-M4	52 (77.6)	54 (80.6)	
M5 (%)	M5	11 (16.4)	2 (3.0)	0.02
	Non-M5	56 (83.6)	65 (97.0)	
M6 (%)	M6	1 (1.5)	1 (1.5)	1
	Non-M6	66 (98.5)	66 (98.5)	
M7 (%)	M7	0 (0.0)	1 (1.5)	1
	Non-M7	67 (100.0)	66 (98.5)	
WBC [mean (*SD*)]		37.54 (44.40)	32.00 (39.79)	0.448
Hemoglobin [mean (*SD*)]		9.51 (1.46)	9.70 (1.38)	0.431
Platelet [mean (*SD*)]		68.27 (57.36)	60.01 (51.91)	0.384
FLT3 mutation (%)	Negative	49 (73.1)	45 (67.2)	0.571
	Positive	18 (26.9)	22 (32.8)	
Activating RAS (%)	Negative	63 (94.0)	63 (94.0)	1
	Positive	4 (6.0)	4 (6.0)	
NPMc (%)	Negative	52 (77.6)	51 (76.1)	1
	Positive	15 (22.4)	16 (23.9)	
IDH1 R172 (%)	Negative	66 (98.5)	66 (98.5)	1
	Positive	1 (1.5)	1 (1.5)	
IDH1 R132 (%)	Negative	61 (91.0)	61 (91.0)	1
	Positive	6 (9.0)	6 (9.0)	
IDH1 R140 (%)	Negative	62 (92.5)	62 (92.5)	1
	Positive	5 (7.5)	5 (7.5)	

**TABLE 2:** AP2M1 expression is associated with multiple clinical and molecular characteristics of AML.

		AP2M1 expression level		
		
		High (*n* = 64)	Low (*n* = 64)	*P*
Gender (%)	FEMALE	29 (43.3)	32 (47.8)	0.729
	MALE	38 (56.7)	35 (52.2)	
Status (%)	Alive	18 (26.9)	30 (44.8)	0.047
	Dead	49 (73.1)	37 (55.2)	
OS [mean (*SD*)]		354.25 (419.13)	758.52 (657.14)	<0.001
M0 (%)	M0	3 (4.5)	11 (16.4)	0.048
	Non-M0	64 (95.5)	56 (83.6)	
M1 (%)	M1	19 (28.4)	13 (19.4)	0.311
	Non-M1	48 (71.6)	54 (80.6)	
M2 (%)	M2	13 (19.4)	17 (25.4)	0.534
	Non-M2	54 (80.6)	50 (74.6)	
M3 (%)	M3	6 (9.0)	8 (11.9)	0.778
	Non-M3	61 (91.0)	59 (88.1)	
M4 (%)	M4	12 (17.9)	16 (23.9)	0.524
	Non-M4	55 (82.1)	51 (76.1)	
M5 (%)	M5	11 (16.4)	2 (3.0)	0.02
	Non-M5	56 (83.6)	65 (97.0)	
M6 (%)	M6	2 (3.0)	0 (0.0)	0.476
	Non-M6	65 (97.0)	67 (100.0)	
M7 (%)	M7	1 (1.5)	0 (0.0)	1
	Non-M7	66 (98.5)	67 (100.0)	
WBC [mean (*SD*)]		40.82 (46.80)	28.72 (36.13)	0.096
Hemoglobin [mean (*SD*)]		9.54 (1.46)	9.67 (1.39)	0.586
Platelet [mean (*SD*)]		53.51 (35.46)	74.78 (67.32)	0.024
FLT3 mutation (%)	Negative	46 (68.7)	48 (71.6)	0.85
	Positive	21 (31.3)	19 (28.4)	
Activating RAS (%)	Negative	64 (95.5)	62 (92.5)	0.715
	Positive	3 (4.5)	5 (7.5)	
NPMc (%)	Negative	52 (77.6)	51 (76.1)	1
	Positive	15 (22.4)	16 (23.9)	
IDH1 R172 (%)	Negative	67 (100.0)	65 (97.0)	0.476
	Positive	0 (0.0)	2 (3.0)	
IDH1 R132 (%)	Negative	63 (94.0)	59 (88.1)	0.364
	Positive	4 (6.0)	8 (11.9)	
IDH1 R140 (%)	Negative	65 (97.0)	59 (88.1)	0.1
	Positive	2 (3.0)	8 (11.9)	

### Prediction of Chemotherapy Effect

**FIGURE 5:**
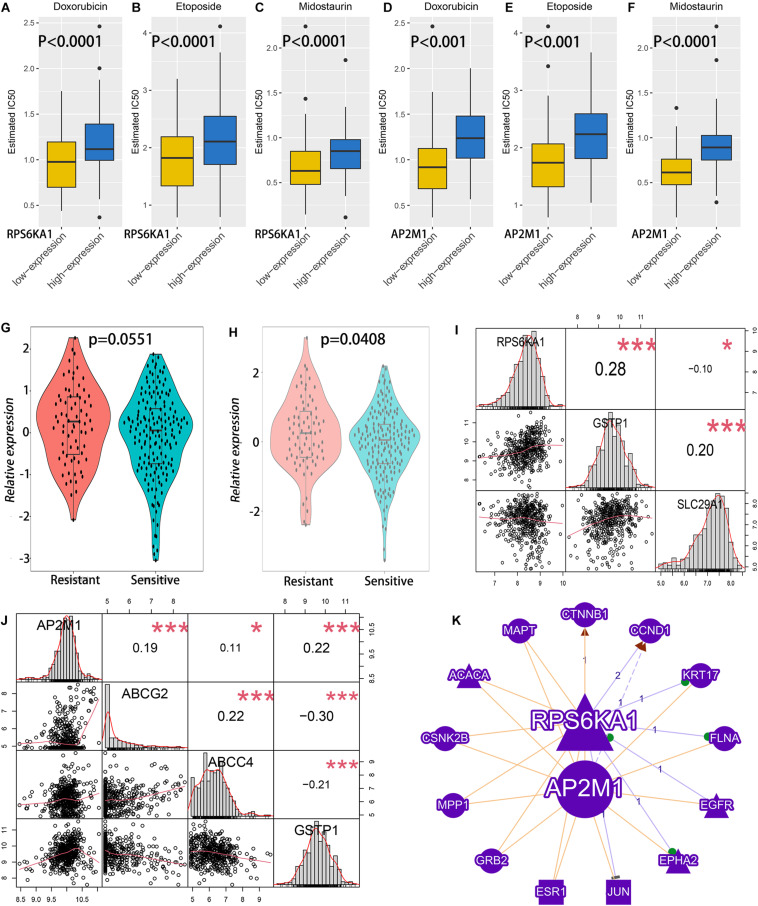
The relationship between RPS6KA1/AP2M1 expression levels and chemotherapy effects of AML patients. **(A)** Sensitivity analysis of doxorubicin in patients with RPS6KA1 high and low expression levels in TCGA-AML. **(B)** Sensitivity analysis of etoposide in patients with RPS6KA1 high and low expression levels in TCGA-AML. **(C)** Sensitivity analysis of midostaurin in patients with RPS6KA1 high and low expression levels in TCGA-AML. **(D)** Sensitivity analysis of doxorubicin in patients with AP2M1 high and low expression levels in TCGA-AML. **(E)** Sensitivity analysis of etoposide in patients with AP2M1 high and low expression levels in TCGA-AML. **(F)** Sensitivity analysis of midostaurin in patients with AP2M1 high and low expression levels in TCGA-AML. **(G)** Analysis of relative expression of RPS6KA1 in the resistance group and sensitive group from GSE106291. **(H)** Analysis of relative expression of AP2M1 in the resistance group and sensitive group from GSE106291. **(I)** Correlations between RPS6KA1 and GSTP1, between RPS6KA1 and SLC29A1 from TCGA-AML. **(J)** Correlations between AP2M1 and ABCG2, between AP2M1 and ABCC4, and between AP2M1 and GSTP1 from TCGA-AML. **(K)** A gene network of RPS6KA1 and AP2M1 with common enzymes and transcription factors by GenCLiP 3.0. *p < 0.05; ***p < 0.001.

The IC50 of common drugs was predicted in different groups by using the pRRophetic algorithm. High RPS6KA1/AP2M1 expression groups had higher IC50 of two chemotherapeutic agents (doxorubicin and etoposide) and one targeted drug (midostaurin, a kind of multitargeted kinase inhibitor) (Figures 5A–F). In addition, both RPS6KA1 and AP2M11 expression levels are significantly elevated in the chemotherapy-resistant group than that of the chemotherapy-sensitive group in GSE106291 (Figures 5G,H). To further explore the possible mechanisms of drug resistance, the correlations between RPS6KA1/AP2M1 and well-known drug-resistance-associated genes were calculated in TCGA-AML, and we found that the expression of RPS6KA1 correlated positively with GSTP1 and negatively with SLC29A1 (Figure 5I). The expression level of AP2M1 was positively associated with ABCG2, ABCC4, and GSTP1 (Figure 5J). Besides, the results of gene network analysis by GenCLiP 3.0 showed that both RPS6KA1 and AP2M1 had a common enzyme and transcription factor (Figure 5K).

### The Relationships Between RPS6KA1 and Leukemia Stem Cell, Between AP2M1 and Leukemia Stem Cell

**FIGURE 6:**
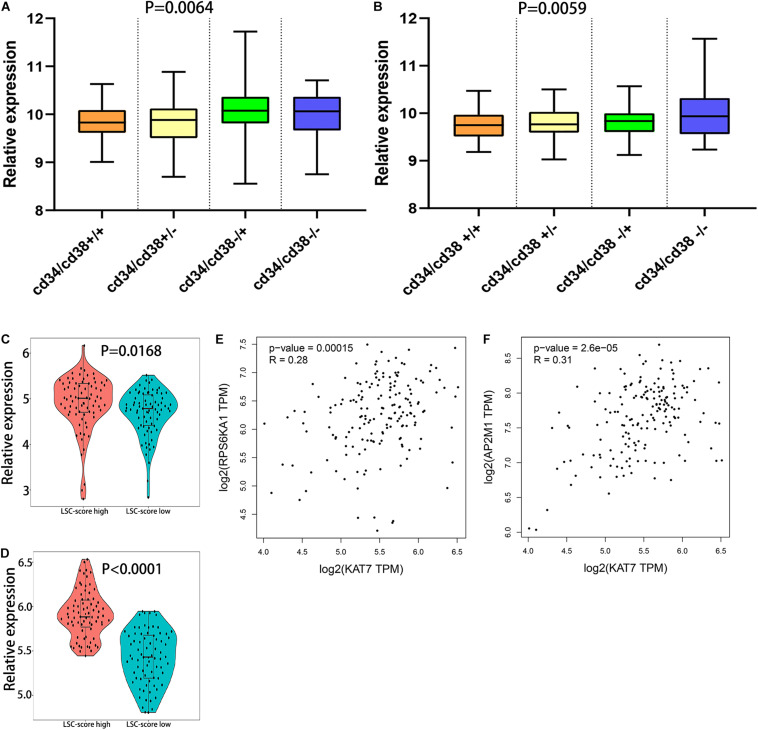
The relationship between the expression level of RPS6KA1/AP2M1 and leukemia stem cells. **(A)** The relative expression of RPS6KA1 in four different groups based on CD34± and CD38± from GSE76008. **(B)** The relative expression of AP2M1 in four different groups based on CD34± and CD38± from GSE76008. **(C)** The relative expression of RPS6KA1 in the high LSC-score group and low LSC-score group. **(D)** The relative expression of AP2M1 in the high LSC-score group and low LSC-score group. **(E)** Gene expression correlation of RPS6KA1 and KAT7 from GEPIA. **(F)** Gene expression correlation of AP2M1 and KAT7 from GEPIA.

Leukemic stem cell (LSC), a kind of classic tumor stem cell, can produce a heterogeneous population of leukemia cells with the ability of self-renewal and differentiation. The surface molecular characteristic of LSC is CD34+/CD38− (Prus and Fibach, 2003; Cao et al., 2019a). The samples of GSE76008 were used to identify the relationships between leukemia stem cell and RPS6KA1/AP2M1 expression. We found that RPS6KA1 expression and AP2M1 expression were obviously different between 4 groups with different characteristics (CD34± and CD38±), separately (*P* = 0.0064, *P* = 0.0059, Figures 6A,B). The RPS6KA1 expression increased in the group with CD34−/CD38+ and induced in the group with CD34+/CD38+; the AP2M1 expression increased in the group with CD34−/CD38− and induced in the group with CD34+/CD38+. Furthermore, RPS6KA1 expression and AP2M1 expression in TCGA-AML increased in the group with a high LSC17 score (LSC17 score, a prognostic factor for AML) (Figures 6C,D) (Ng et al., 2016). The correlation between the expression levels of RPS6KA1/AP2M1 and KAT7 (one key molecular in LSC) was computed, and we demonstrated that the expression levels of RPS6KA1 and AP2M1 are significantly positive correlated with KAT7 expression (Figures 6E,F).

### Relationship Between RPS6KA1/AP2M1 and the Immune Microenvironment in AML

**FIGURE 7:**
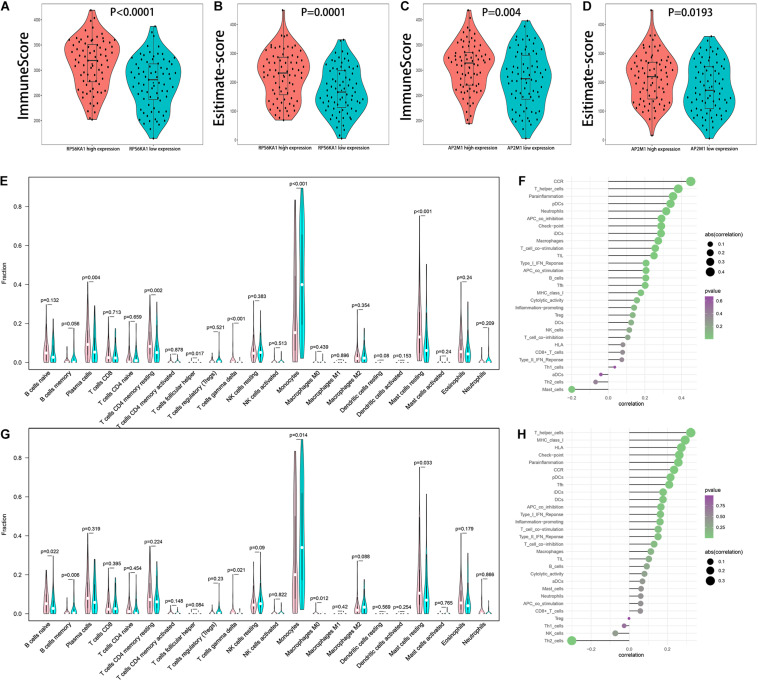
Relationship between RPS6KA1/AP2M1 and immune microenvironment in AML. **(A)** Differences in immune score between AML patients with RPS6KA1 high expression and RPS6KA1 low expression. **(B)** Differences in estimate score between AML patients with RPS6KA1 high expression and RPS6KA1 low expression. **(C)** Differences in immune score between AML patients with AP2M1 high expression and AP2M1 low expression. **(D)** Differences in estimate score between AML patients with AP2M1 high expression and AP2M1 low expression. **(E)** The comparison of immune infiltration level between the RPS6KA1 high expression group and RPS6KA1 low expression group based on CIBERSORT. **(F)** Correlation between RPS6KA1 and immune-infiltrating cells based on the ssGSEA approach. **(G)** The comparison of immune infiltration level between the AP2M1 high expression group and RPS6KA1 low expression group based on CIBERSORT. **(H)** Correlation between AP2M1 and immune-infiltrating cells based on the ssGSEA approach.

Comparing immune score, estimate score, and tumor purity of high and low RPS6KA1/AP2M1 expression groups, AML patients in high RPS6KA1/AP2M1 expression groups had a higher immune score, higher estimate score, and lower tumor purity (Figures 7A–D and Supplementary Figure 2). Besides, the composition of 22 immune cells in each patient of TCGA-AML was estimated by CIBERSORT. Comparing the composition of the immune cells of high and low RPS6KA1/AP2M1 expression groups, the patients in the high RPS6KA1 expression group had a higher proportion of plasma cells, resting memory CD4(+) T cells, follicular helper T cells, γδ T cells, and resting mast cells, but a lower proportion of monocytes (Figure 7E); the patients in the high AP2M1 expression group had a higher proportion of naive B cells, γδ T cells, M0 macrophages, and resting mast cells, but a lower proportion of memory B cells and monocytes (Figure 7G). Based on the results of ssGSEA, both the expression levels of RPS6KA1 and AP2M1 were significantly positively associated with chemokine receptor (CCR), checkpoint, T helper cells, and immature dendritic cells (iDCs) (Figures 7F,H).

### Mechanisms Involved in Dysregulation of RPS6KA1/AP2M1 Expression

**FIGURE 8:**
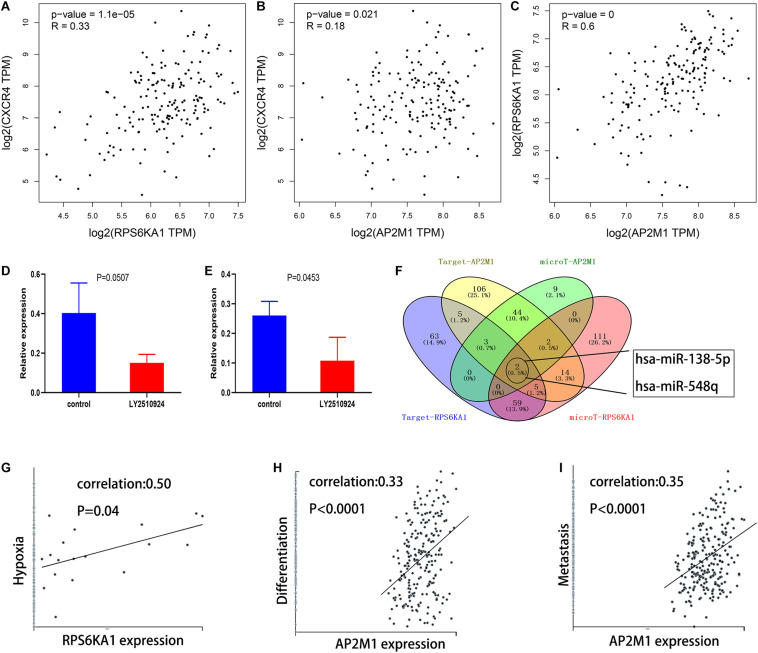
Mechanisms involved in dysregulation of RPS6KA1/AP2M1 expression. **(A)** Gene expression correlation of RPS6KA1 and CXCR4 from GEPIA. **(B)** Gene expression correlation of AP2M1 and CXCR4 from GEPIA. **(C)** Gene expression correlation of RPS6KA1 and AP2M1 from GEPIA. **(D)** The relative expression of RPS6KA1 in the control group and CXCR4 inhibitor group from GSE64623. **(E)** The relative expression of AP2M1 in the control group and CXCR4 inhibitor group from GSE64623. **(F)** Identification of the common targeted miRNAs of RPS6KA1 and AP2M1 from microT-CDS and TargetScan. **(G)** Correlation between RPS6KA1 expression and functional states of hypoxia based on CancerSEA. **(H)** Correlation between AP2M1 expression and functional states of differentiation based on CancerSEA. **(I)** Correlation between AP2M1 expression and functional states of metastasis based on CancerSEA.

**FIGURE 9:**
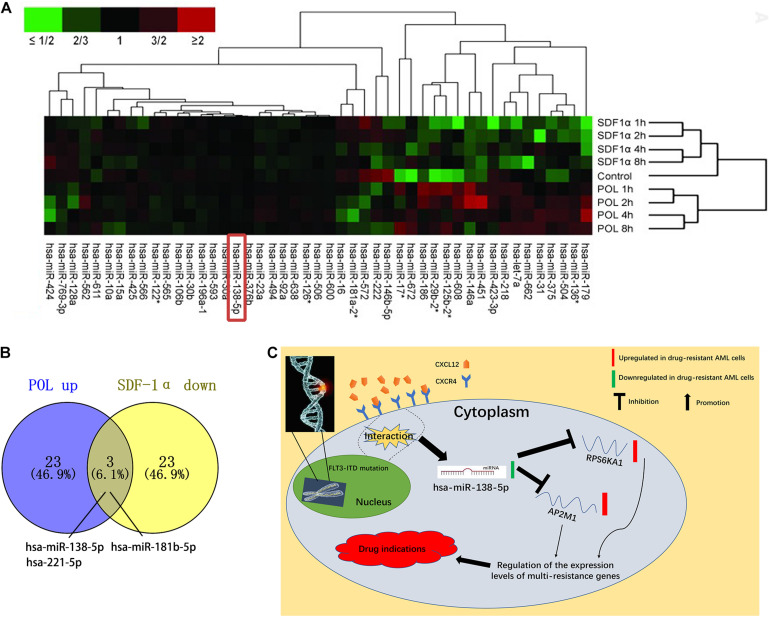
miR-138-5p plays an important role in AML chemoresistance, as an intermediate molecule. **(A)** Heat map of differentially expressed miRNAs in different groups. **(B)** The intersection of differentially expressed miRNAs with SDF-1 α downregulation and POL6326 upregulation, which is regulated by the CXCL12/CXCR4 axis. **(C)** Model of the miR-138-5p/RPS6KA1-AP2M1 network and its expression and potential roles in AML chemoresistance.

Based on the GEPIA database, we demonstrated that RPS6KA1 expression was positively correlated with AP2M1 expression (*P* < 0.0001), and there were positive correlations between the expression levels of RPS6KA1 and CXCR4 (*P* < 0.0001) and between the expression levels of AP2M1 and CXCR4 (*P* = 0.021) (Figures 8A–C). To further clarify the relationship between the CXCL12/CXCR4 axis and RPS6KA1/AP2M1, GSE64623, containing the control group (OCI-AML3 cells) and treatment group (OCI-AML3 cells treated by LY2510294, a kind of inhibitor for CXCR4), was used to explore the expression levels of RPS6KA1/AP2M1 in different groups. The expression level of RPS6KA1 decreased after the treatment with LY2510294 in OCI-AML3 cells (*P* = 0.0507), and the expression level of AP2M1 also decreased in the treatment group (*P* = 0.0453) (Figures 8D,E). According to the predicted results of TargetScan and microT-CDS, we found that RPS6KA1 and AP2M1 are all regulated by common miRNAs (miR-138-5p and miR-548q) (Figure 8F). Moreover, the results of CancerSEA revealed that RPS6KA1 was positively associated with hypoxia and AP2M1 was associated with differentiation and metastasis (Figures 8G–I). In order to identify the relationship between upstream potential miRNA and RPS6KA1/AP2M1, “limma” package in R was applied to clarify the differentially expressed miRNAs between the control group and the treatment group in GSE44828, and the results suggested that the CXCL12/CXCR4 axis regulated the miR-138-5p expression levels in AML cells (Figures 9A,B). Taken together, the interaction of FLT3-ITD mutation and the CXCL12/CXCR4 axis activates the PI3K–Akt–mTOR pathway, and downregulation of hsa-miR-138-5p causes overexpression of RPS6KA1 and AP2M1, regulating the expression levels of multi-resistance genes resulting in drug indications (Figure 9C).

## Discussion

FLT3-ITD is related to chemoresistance and poor prognosis in the patients with AML (Jetani et al., 2018). The CXCR4/CXCL12 axis plays the important part in the interactions between the microenvironment in bone marrow and AML cell, which have already been linked to chemoresistance and relapse of AML (Zhang et al., 2019; Ladikou et al., 2020). As the essential indicators in AML progression, there is an intimate connection between FLT3-ITD mutation and the CXCR4/CXCL12 axis with multiple interactions (Fukuda et al., 2005). For example, FLT3-ITD mutation upregulates the expression levels of STAT5 (a regulatory factor for CXCR4 mRNA level), causing an increase in CXCR4 expression on the cell surface in AML (Cao et al., 2019b). Besides, LY2510924, a CXCR4 inhibition, could significantly increase the effect of FLT3 inhibitors in the AML cells with FLT3-ITD mutation (Kim et al., 2020). Identifying the common downstream cellular mechanisms of FLT3-ITD and CXCR4-CXCL12 axis helps to further understand the interactions between them from another aspect.

In this study, RPS6KA1 and AP2M1 were identified as prognostic indicators, and they were significantly related to chemoresistance of AML. According to the results of clinical analysis of characteristics, we demonstrated that the AML patients with M5 mostly showed higher RPS6KA1 and AP2M1 expression. RPS6KA1 expression and AP2M1 expression were significantly associated with the status of LSC. Recent research reveals that KAT7 protein, as the MYST acetyltransferase, is critical for the maintenance in LSC (Sauer et al., 2015; MacPherson et al., 2020). The significant positive correlations between RPS6KA1 and KAT7 and between AP2M1 and KAT7 showed that RPS6KA1 and AP2M1 might influence the survival of LSC.

We discovered that RPS6KA1 expression and AP2M1 expression are negatively associated with OS in AML based on TCGA-AML. RPS6KA1 belongs to the family of the ribosomal protein S6 kinase (RPS6K) (Li et al., 2012; Czaplinska et al., 2018). The RPS6K family is reported to be involved in a lot of pathways, which are important for tumor occurrence and progression (Hajj et al., 2020; Lai et al., 2020; Yang et al., 2020). A new study indicates that RPS6KA1 is a potential target of new treatment strategies for drug-resistant AML patients with FLT3-ITD mutation, especially combining PI3K, PIM, or Bcl-2 family members (Watanabe et al., 2019). As for AP2M1, its role in gallbladder cancer, mucoepidermoid carcinoma, liver cancer, and adenoid cystic carcinoma was generally clear (Cho et al., 2019; Song et al., 2020; Wu et al., 2020), but it is seldom reported how AP2M1 is involved in AML progression.

According to the results of GSVA in four independent datasets, we demonstrated that the PI3K–AKT–mTOR pathway was significantly activated in FLT3-ITD-positive AML patients. The PI3K–AKT–mTOR pathway participates in various biological processes, including DNA replication, survival, translation, and chemoresistance (Bertacchini et al., 2014, 2015; Nepstad et al., 2018). Previous studies have revealed that 60–80 percent of AML patients had constitutive activation of the PI3K–Akt–mTOR pathway (Herschbein and Liesveld, 2018). Deng demonstrated that BEZ235, a strong inhibitor for the PI3K–AKT–mTOR pathway, could effectively improve the effects of chemotherapy and prevent proliferation and migration of AML cells (Deng et al., 2017). Though the clinical practical applications of PI3K–AKT–mTOR inhibitors in AML patients were not satisfying, the combinations of PI3K–Akt–mTOR pathway inhibitors and other pharmaceuticals showed good results (Vachhani et al., 2014; Nepstad et al., 2020). For example, the application of the combined Midostaurin may have double effects of inhibiting FLT3 activation, and FLT3-ITD-positive patients could get greater benefit (Stone et al., 2017; Nepstad et al., 2020). Information of the PI3K–AKT–mTOR pathway in AML is still not enough, particularly the role in AML chemoresistance. Further research, especially the regulatory molecular mechanisms, is badly needed to open up a new phase for the application of PI3K–AKT–mTOR inhibitors.

Based on the predicted results of TargetScan and microT-CDS, miR-138-5p was selected as the common miRNA target for RPS6KA1 and AP2M1, and we found that miR-138-5p was regulated by the CXCL12/CXCR4 axis from another gene expression profile. Activation of the CXCL12/CXCR4 axis leads to enhanced chemotaxis, transendothelial migration, and invasion; hence, it was supposed that miR-138-5p is essential for AML chemoresistance, as an intermediate molecular associating CXCL12/CXCR4 axis with multidrug resistance genes, and we conducted a literature review of miR-138-5p. Previous studies have shown its important role as an anti-oncogene in some cancers, including non-small cell lung cancer, glioma, and gastric cancer (Gao et al., 2014; Bai et al., 2019; He et al., 2019; Ning et al., 2019). Besides, miR-138-5p had been identified to affect chemotherapy sensitivity through induction of apoptosis, inhibition of EMT, and inhibition of DNA repair (Gao et al., 2014; Yu et al., 2015). We supposed that miR-138-5p might regulate multi-resistance gene expression by affecting the expression levels of RPS6KA1 and AP2M1. However, the exact biological role of miR-138-5p in AML drug resistance was not known. In the future, we will further explore the function of miR-138-5p for AML *in vivo* and *in vitro* and investigate the mechanism of the low expression of miR-138-5p.

In summary, RPS6KA1 and AP2M1 can be used prognosis indicators of AML, and high expression levels of RPS6KA1 and AP2M1 were related to chemoresistance and bad prognosis in AML patients. Moreover, the miR-138-5p/RPS6KA1-AP2M1 axis was successfully identified, which would provide new clues to solve the clinical resistance issue in AML, and the constituents in this axis probably become effective therapeutic targets in treatment of AML patients in the future.

## Data Availability Statement

Publicly available datasets were analyzed in this study. This data can be found here: https://www.cancer.gov/about-nci/organization/ccg/research/structural-genomics/.

## Author Contributions

X-LR and SL conceived and designed the study. D-HY and X-PL performed the analysis procedures. D-HY, JY, CC, and X-PL analyzed the results. X-PL, X-LR, and SL contributed analysis tools. D-HY and X-LR contributed to the writing of the manuscript. All authors reviewed the manuscript.

## Conflict of Interest

The authors declare that the research was conducted in the absence of any commercial or financial relationships that could be construed as a potential conflict of interest.

## Footnotes

1 https://tcga.xenahubs.net

2 https://www.ncbi.nlm.nih.gov/geo/

3 https://www.gsea-msigdb.org/gsea/msigdb

4 http://ualcan.path.uab.edu/index.html

5 www.cancerrxgene.org/

6 http://ci.smu.edu.cn/genclip3/analysis.php

7 http://gepia.cancer-pku.cn/detail.php

8 https://cibersort.stanford.edu

9 http://biocc.hrbmu.edu.cn/CancerSEA/home.jsp

10 www.targetscan.org/

11 http://www.microrna.gr/webServer

12 https://www.gsea-msigdb.org/gsea/msigdb

13 https://david.ncifcrf.gov/

## Supplementary Material

Supplementary Figure 1 Clustering dendrograms of genes based on a dissimilarity measure (1-TOM).

Supplementary Figure 2 Differences in tumor purity between AML patients with RPS6KA1/AP2M1 high and low expression groups.

Supplementary Table 1 Gene and miRNA expression microarray datasets related to AML.

Supplementary Table 2 A summary of the GSVA of GSE6091.

Supplementary Table 3 A summary of the GSVA of GSE10358.

Supplementary Table 4 A summary of the GSVA of GSE15434.

Supplementary Table 5 A summary of the GSVA of GSE61804.

Supplementary Table 6 The enriched terms of BP for the genes in green module.

Supplementary Table 7 The enriched terms of CC for the genes in green module.

Supplementary Table 8 The enriched terms of MF for the genes in green module.
