# Supplementary material for: Dysregulation of miR-138-5p/RPS6KA1-AP2M1 Is Associated With Poor Prognosis in AML
Source: Front Cell Dev Biol. 2021 Feb 26;9:641629. doi: 10.3389/fcell.2021.641629 (PMC7959750; doi:10.3389/fcell.2021.641629)
Supplement: Supplementary Figure 1 — Clustering dendrograms of genes based on a dissimilarity measure (1-TOM). [file Data_Sheet_1.ZIP › supplemental materials/Table S1.docx]

**Table S1:** **Gene and miRNA expression microarray datasets related to AML.**

|  | Accession number of the dataset | Platform | Organism | Experiment type |
| --- | --- | --- | --- | --- |
| mRNAs | GSE6891 | GPL570 | Homo sapiens | Expression profiling by array |
|  | GSE10358 | GPL570 | Homo sapiens | Expression profiling by array |
|  | GSE15434 | GPL570 | Homo sapiens | Expression profiling by array |
|  | GSE61804 | GPL570 | Homo sapiens | Expression profiling by array |
|  | GSE64623 | GPL10558 | Homo sapiens | Expression profiling by array |
|  | GSE76004 | GPL21241 | Homo sapiens | Expression profiling by array |
|  | GSE76008 | GPL10558 | Homo sapiens | Expression profiling by array |
|  | GSE106291 | GPL18460 | Homo sapiens | Expression profiling by high throughput sequencing |
| miRNAs | GSE44828 | GPL16744 | Homo sapiens | Non-coding RNA profiling by array |

Note. miRNA: microRNA. AML: acute myeloid leukemia.
