# Supplementary material for: Dysregulation of miR-138-5p/RPS6KA1-AP2M1 Is Associated With Poor Prognosis in AML
Source: Front Cell Dev Biol. 2021 Feb 26;9:641629. doi: 10.3389/fcell.2021.641629 (PMC7959750; doi:10.3389/fcell.2021.641629)
Supplement: Supplementary Figure 1 — Clustering dendrograms of genes based on a dissimilarity measure (1-TOM). [file Data_Sheet_1.ZIP › supplemental materials/Table S2.docx]

**Table S2. A summary of the GSVA of GSE6091.**

| Term | logFC | t | P.Value |
| --- | --- | --- | --- |
| HALLMARK_PI3K_AKT_MTOR_SIGNALING | 0.108643 | 4.955999 | 1.01E-06 |
| HALLMARK_FATTY_ACID_METABOLISM | 0.085488 | 4.348025 | 1.69E-05 |
| HALLMARK_GLYCOLYSIS | 0.088723 | 4.262944 | 2.44E-05 |
| HALLMARK_MYC_TARGETS_V2 | 0.10455 | 2.699097 | 0.007206 |
| HALLMARK_MTORC1_SIGNALING | 0.085595 | 2.640712 | 0.008552 |
| HALLMARK_DNA_REPAIR | 0.063258 | 2.47308 | 0.013752 |
| HALLMARK_MYC_TARGETS_V1 | 0.091386 | 2.316854 | 0.020946 |
| HALLMARK_IL2_STAT5_SIGNALING | 0.034545 | 2.214222 | 0.027299 |
| HALLMARK_ADIPOGENESIS | 0.040025 | 2.189495 | 0.029058 |
| HALLMARK_P53_PATHWAY | 0.02327 | 1.488776 | 0.137225 |
| HALLMARK_G2M_CHECKPOINT | 0.041473 | 1.451249 | 0.147386 |
| HALLMARK_OXIDATIVE_PHOSPHORYLATION | 0.054667 | 1.407612 | 0.159915 |
| HALLMARK_UNFOLDED_PROTEIN_RESPONSE | 0.029302 | 1.159308 | 0.246926 |
| HALLMARK_PROTEIN_SECRETION | 0.027142 | 0.960513 | 0.337297 |
| HALLMARK_PEROXISOME | 0.016425 | 0.808832 | 0.419026 |
| HALLMARK_CHOLESTEROL_HOMEOSTASIS | 0.010529 | 0.438755 | 0.661043 |
| HALLMARK_XENOBIOTIC_METABOLISM | 0.005483 | 0.426983 | 0.669589 |
| HALLMARK_APOPTOSIS | 0.007355 | 0.308636 | 0.757737 |
| HALLMARK_E2F_TARGETS | 0.007659 | 0.169417 | 0.865542 |
| HALLMARK_UV_RESPONSE_DN | -0.00134 | -0.06965 | 0.944503 |
| HALLMARK_ANDROGEN_RESPONSE | -0.007 | -0.42003 | 0.674658 |
| HALLMARK_BILE_ACID_METABOLISM | -0.00637 | -0.43551 | 0.663396 |
| HALLMARK_REACTIVE_OXIGEN_SPECIES_PATHWAY | -0.01553 | -0.54116 | 0.588655 |
| HALLMARK_ANGIOGENESIS | -0.02373 | -0.69353 | 0.488323 |
| HALLMARK_INFLAMMATORY_RESPONSE | -0.03092 | -1.10486 | 0.269792 |
| HALLMARK_TGF_BETA_SIGNALING | -0.02919 | -1.21675 | 0.224317 |
| HALLMARK_ESTROGEN_RESPONSE_LATE | -0.02097 | -1.24242 | 0.214708 |
| HALLMARK_KRAS_SIGNALING_DN | -0.0178 | -1.25438 | 0.210335 |
| HALLMARK_SPERMATOGENESIS | -0.02435 | -1.79181 | 0.073814 |
| HALLMARK_PANCREAS_BETA_CELLS | -0.03382 | -1.90171 | 0.057828 |
| HALLMARK_INTERFERON_GAMMA_RESPONSE | -0.0738 | -2.16944 | 0.030556 |
| HALLMARK_ESTROGEN_RESPONSE_EARLY | -0.03222 | -2.26809 | 0.023782 |
| HALLMARK_MITOTIC_SPINDLE | -0.04158 | -2.38278 | 0.017584 |
| HALLMARK_UV_RESPONSE_UP | -0.04894 | -2.44933 | 0.014681 |
| HALLMARK_HEDGEHOG_SIGNALING | -0.06251 | -2.48829 | 0.013185 |
| HALLMARK_KRAS_SIGNALING_UP | -0.03871 | -2.59124 | 0.009864 |
| HALLMARK_HYPOXIA | -0.05171 | -2.743 | 0.006323 |
| HALLMARK_COAGULATION | -0.0554 | -2.84334 | 0.004661 |
| HALLMARK_ALLOGRAFT_REJECTION | -0.07038 | -2.84837 | 0.004589 |
| HALLMARK_WNT_BETA_CATENIN_SIGNALING | -0.06599 | -2.93486 | 0.003502 |
| HALLMARK_HEME_METABOLISM | -0.11697 | -2.95473 | 0.003288 |
| HALLMARK_TNFA_SIGNALING_VIA_NFKB | -0.11292 | -3.33087 | 0.000935 |
| HALLMARK_NOTCH_SIGNALING | -0.0922 | -3.50022 | 0.00051 |
| HALLMARK_IL6_JAK_STAT3_SIGNALING | -0.10108 | -3.60588 | 0.000345 |
| HALLMARK_EPITHELIAL_MESENCHYMAL_TRANSITION | -0.06181 | -3.73528 | 0.000211 |
| HALLMARK_COMPLEMENT | -0.09172 | -3.82512 | 0.000149 |
| HALLMARK_INTERFERON_ALPHA_RESPONSE | -0.15368 | -4.11947 | 4.50E-05 |
| HALLMARK_APICAL_SURFACE | -0.10665 | -4.2778 | 2.29E-05 |
| HALLMARK_APICAL_JUNCTION | -0.06956 | -4.63974 | 4.54E-06 |
| HALLMARK_MYOGENESIS | -0.07156 | -4.89636 | 1.35E-06 |
