# Supplementary material for: Dysregulation of miR-138-5p/RPS6KA1-AP2M1 Is Associated With Poor Prognosis in AML
Source: Front Cell Dev Biol. 2021 Feb 26;9:641629. doi: 10.3389/fcell.2021.641629 (PMC7959750; doi:10.3389/fcell.2021.641629)
Supplement: Supplementary Figure 1 — Clustering dendrograms of genes based on a dissimilarity measure (1-TOM). [file Data_Sheet_1.ZIP › supplemental materials/Table S3.docx]

**Table S2. A summary of the GSVA of GSE10358.**

| Term | logFC | t | P.Value |
| --- | --- | --- | --- |
| HALLMARK_GLYCOLYSIS | 0.104895 | 3.047412 | 0.002631 |
| HALLMARK_DNA_REPAIR | 0.121671 | 2.828023 | 0.005176 |
| HALLMARK_PI3K_AKT_MTOR_SIGNALING | 0.085109 | 2.209787 | 0.028293 |
| HALLMARK_MYC_TARGETS_V2 | 0.11278 | 1.699468 | 0.090838 |
| HALLMARK_MYC_TARGETS_V1 | 0.096313 | 1.537397 | 0.125829 |
| HALLMARK_FATTY_ACID_METABOLISM | 0.052918 | 1.514002 | 0.131657 |
| HALLMARK_UNFOLDED_PROTEIN_RESPONSE | 0.066907 | 1.473938 | 0.142123 |
| HALLMARK_OXIDATIVE_PHOSPHORYLATION | 0.068736 | 1.265297 | 0.207286 |
| HALLMARK_MTORC1_SIGNALING | 0.061616 | 1.15584 | 0.249171 |
| HALLMARK_CHOLESTEROL_HOMEOSTASIS | 0.04293 | 1.126956 | 0.261156 |
| HALLMARK_ADIPOGENESIS | 0.035794 | 1.126777 | 0.261232 |
| HALLMARK_E2F_TARGETS | 0.070133 | 0.984914 | 0.325896 |
| HALLMARK_PROTEIN_SECRETION | 0.040523 | 0.861844 | 0.38984 |
| HALLMARK_G2M_CHECKPOINT | 0.037744 | 0.806538 | 0.420923 |
| HALLMARK_ANDROGEN_RESPONSE | 0.025484 | 0.754238 | 0.451623 |
| HALLMARK_PEROXISOME | 0.023141 | 0.743873 | 0.457855 |
| HALLMARK_XENOBIOTIC_METABOLISM | 0.012293 | 0.561641 | 0.57501 |
| HALLMARK_SPERMATOGENESIS | 0.012933 | 0.543887 | 0.587145 |
| HALLMARK_APOPTOSIS | 0.013535 | 0.362794 | 0.717154 |
| HALLMARK_TGF_BETA_SIGNALING | 0.016895 | 0.354572 | 0.723296 |
| HALLMARK_P53_PATHWAY | 0.008289 | 0.263464 | 0.792473 |
| HALLMARK_REACTIVE_OXIGEN_SPECIES_PATHWAY | 0.011181 | 0.248975 | 0.803644 |
| HALLMARK_IL2_STAT5_SIGNALING | 0.005547 | 0.217769 | 0.827839 |
| HALLMARK_ESTROGEN_RESPONSE_LATE | 0.00098 | 0.034325 | 0.972653 |
| HALLMARK_HEDGEHOG_SIGNALING | -0.0014 | -0.03084 | 0.975432 |
| HALLMARK_ANGIOGENESIS | -0.0068 | -0.10494 | 0.916534 |
| HALLMARK_COAGULATION | -0.00562 | -0.15573 | 0.876405 |
| HALLMARK_UV_RESPONSE_UP | -0.00581 | -0.18203 | 0.855747 |
| HALLMARK_TNFA_SIGNALING_VIA_NFKB | -0.01624 | -0.29804 | 0.765989 |
| HALLMARK_APICAL_SURFACE | -0.02576 | -0.63412 | 0.526749 |
| HALLMARK_UV_RESPONSE_DN | -0.02257 | -0.68653 | 0.4932 |
| HALLMARK_PANCREAS_BETA_CELLS | -0.0388 | -0.96807 | 0.334219 |
| HALLMARK_KRAS_SIGNALING_DN | -0.0284 | -0.96909 | 0.333711 |
| HALLMARK_HYPOXIA | -0.03647 | -1.1687 | 0.24396 |
| HALLMARK_EPITHELIAL_MESENCHYMAL_TRANSITION | -0.04648 | -1.4497 | 0.148759 |
| HALLMARK_MYOGENESIS | -0.04215 | -1.48733 | 0.138556 |
| HALLMARK_WNT_BETA_CATENIN_SIGNALING | -0.05941 | -1.52626 | 0.128577 |
| HALLMARK_NOTCH_SIGNALING | -0.06555 | -1.55209 | 0.122275 |
| HALLMARK_MITOTIC_SPINDLE | -0.05042 | -1.56844 | 0.118411 |
| HALLMARK_APICAL_JUNCTION | -0.03728 | -1.62773 | 0.105209 |
| HALLMARK_INFLAMMATORY_RESPONSE | -0.06946 | -1.68142 | 0.094293 |
| HALLMARK_BILE_ACID_METABOLISM | -0.05288 | -1.9253 | 0.055658 |
| HALLMARK_ESTROGEN_RESPONSE_EARLY | -0.05631 | -2.18028 | 0.030443 |
| HALLMARK_HEME_METABOLISM | -0.12985 | -2.4587 | 0.014823 |
| HALLMARK_IL6_JAK_STAT3_SIGNALING | -0.1204 | -2.68817 | 0.007811 |
| HALLMARK_ALLOGRAFT_REJECTION | -0.11494 | -3.25139 | 0.001355 |
| HALLMARK_KRAS_SIGNALING_UP | -0.07854 | -3.27572 | 0.001249 |
| HALLMARK_INTERFERON_GAMMA_RESPONSE | -0.16833 | -3.36542 | 0.000922 |
| HALLMARK_COMPLEMENT | -0.14456 | -4.021 | 8.30E-05 |
| HALLMARK_INTERFERON_ALPHA_RESPONSE | -0.24137 | -4.07652 | 6.67E-05 |
