# Supplementary material for: Dysregulation of miR-138-5p/RPS6KA1-AP2M1 Is Associated With Poor Prognosis in AML
Source: Front Cell Dev Biol. 2021 Feb 26;9:641629. doi: 10.3389/fcell.2021.641629 (PMC7959750; doi:10.3389/fcell.2021.641629)
Supplement: Supplementary Figure 1 — Clustering dendrograms of genes based on a dissimilarity measure (1-TOM). [file Data_Sheet_1.ZIP › supplemental materials/Table S4.docx]

**Table S4. A summary of the GSVA of GSE15434.**

| Term | logFC | t | P.Value |
| --- | --- | --- | --- |
| HALLMARK_PROTEIN_SECRETION | 0.10636 | 3.419614 | 0.000729 |
| HALLMARK_TGF_BETA_SIGNALING | 0.107011 | 3.155185 | 0.001795 |
| HALLMARK_MYC_TARGETS_V1 | 0.140318 | 3.097684 | 0.002167 |
| HALLMARK_PI3K_AKT_MTOR_SIGNALING | 0.07483 | 2.529287 | 0.012029 |
| HALLMARK_APOPTOSIS | 0.070447 | 2.371207 | 0.018469 |
| HALLMARK_DNA_REPAIR | 0.071794 | 2.132206 | 0.03394 |
| HALLMARK_UV_RESPONSE_DN | 0.045424 | 1.978567 | 0.048934 |
| HALLMARK_MITOTIC_SPINDLE | 0.042044 | 1.954225 | 0.051761 |
| HALLMARK_ADIPOGENESIS | 0.041999 | 1.729708 | 0.084885 |
| HALLMARK_P53_PATHWAY | 0.037477 | 1.64647 | 0.100891 |
| HALLMARK_OXIDATIVE_PHOSPHORYLATION | 0.067213 | 1.602784 | 0.110213 |
| HALLMARK_UNFOLDED_PROTEIN_RESPONSE | 0.039199 | 1.258873 | 0.209221 |
| HALLMARK_IL2_STAT5_SIGNALING | 0.025226 | 1.245193 | 0.214197 |
| HALLMARK_MTORC1_SIGNALING | 0.050096 | 1.21724 | 0.224631 |
| HALLMARK_G2M_CHECKPOINT | 0.039289 | 1.139498 | 0.255558 |
| HALLMARK_ANDROGEN_RESPONSE | 0.02708 | 1.109071 | 0.268438 |
| HALLMARK_MYC_TARGETS_V2 | 0.035762 | 0.727348 | 0.467676 |
| HALLMARK_NOTCH_SIGNALING | 0.009359 | 0.275193 | 0.783389 |
| HALLMARK_GLYCOLYSIS | 0.003877 | 0.132306 | 0.894846 |
| HALLMARK_FATTY_ACID_METABOLISM | 0.002287 | 0.084983 | 0.932341 |
| HALLMARK_PANCREAS_BETA_CELLS | -0.00291 | -0.09228 | 0.926546 |
| HALLMARK_BILE_ACID_METABOLISM | -0.00287 | -0.13019 | 0.896519 |
| HALLMARK_INTERFERON_GAMMA_RESPONSE | -0.01004 | -0.24966 | 0.803051 |
| HALLMARK_REACTIVE_OXIGEN_SPECIES_PATHWAY | -0.02546 | -0.65496 | 0.513079 |
| HALLMARK_ALLOGRAFT_REJECTION | -0.02383 | -0.83847 | 0.402546 |
| HALLMARK_IL6_JAK_STAT3_SIGNALING | -0.03178 | -0.95092 | 0.342541 |
| HALLMARK_INTERFERON_ALPHA_RESPONSE | -0.04566 | -1.0746 | 0.283562 |
| HALLMARK_WNT_BETA_CATENIN_SIGNALING | -0.04007 | -1.12749 | 0.26059 |
| HALLMARK_E2F_TARGETS | -0.05963 | -1.22212 | 0.222786 |
| HALLMARK_PEROXISOME | -0.03638 | -1.34867 | 0.178632 |
| HALLMARK_CHOLESTEROL_HOMEOSTASIS | -0.04117 | -1.35795 | 0.175672 |
| HALLMARK_UV_RESPONSE_UP | -0.03722 | -1.48118 | 0.139785 |
| HALLMARK_XENOBIOTIC_METABOLISM | -0.03017 | -1.59105 | 0.112829 |
| HALLMARK_ESTROGEN_RESPONSE_LATE | -0.03681 | -1.68079 | 0.09402 |
| HALLMARK_ESTROGEN_RESPONSE_EARLY | -0.03402 | -1.75658 | 0.080182 |
| HALLMARK_ANGIOGENESIS | -0.08641 | -1.78179 | 0.075967 |
| HALLMARK_SPERMATOGENESIS | -0.03594 | -1.88149 | 0.061038 |
| HALLMARK_TNFA_SIGNALING_VIA_NFKB | -0.08186 | -1.88253 | 0.060895 |
| HALLMARK_COAGULATION | -0.0499 | -1.93461 | 0.054137 |
| HALLMARK_INFLAMMATORY_RESPONSE | -0.08008 | -2.31397 | 0.02146 |
| HALLMARK_KRAS_SIGNALING_UP | -0.05261 | -2.52457 | 0.012188 |
| HALLMARK_APICAL_JUNCTION | -0.06024 | -2.59142 | 0.010106 |
| HALLMARK_COMPLEMENT | -0.08062 | -2.78727 | 0.005713 |
| HALLMARK_HEDGEHOG_SIGNALING | -0.09813 | -2.90891 | 0.003945 |
| HALLMARK_EPITHELIAL_MESENCHYMAL_TRANSITION | -0.07799 | -3.34402 | 0.000949 |
| HALLMARK_KRAS_SIGNALING_DN | -0.08776 | -3.35415 | 0.000916 |
| HALLMARK_HEME_METABOLISM | -0.17618 | -3.82132 | 0.000167 |
| HALLMARK_APICAL_SURFACE | -0.13357 | -3.8776 | 0.000134 |
| HALLMARK_HYPOXIA | -0.08661 | -3.94181 | 0.000104 |
| HALLMARK_MYOGENESIS | -0.12767 | -4.17377 | 4.11E-05 |
