# Supplementary material for: Dysregulation of miR-138-5p/RPS6KA1-AP2M1 Is Associated With Poor Prognosis in AML
Source: Front Cell Dev Biol. 2021 Feb 26;9:641629. doi: 10.3389/fcell.2021.641629 (PMC7959750; doi:10.3389/fcell.2021.641629)
Supplement: Supplementary Figure 1 — Clustering dendrograms of genes based on a dissimilarity measure (1-TOM). [file Data_Sheet_1.ZIP › supplemental materials/Table S5.docx]

**Table S5. A summary of the GSVA of GSE61804.**

| Term | logFC | t | P.Value |
| --- | --- | --- | --- |
| HALLMARK_TGF_BETA_SIGNALING | 0.120269 | 2.851232 | 0.004674 |
| HALLMARK_OXIDATIVE_PHOSPHORYLATION | 0.123498 | 2.42182 | 0.016069 |
| HALLMARK_NOTCH_SIGNALING | 0.08714 | 2.327214 | 0.020656 |
| HALLMARK_MYC_TARGETS_V1 | 0.113231 | 2.296154 | 0.022394 |
| HALLMARK_PI3K_AKT_MTOR_SIGNALING | 0.071138 | 2.215339 | 0.027529 |
| HALLMARK_GLYCOLYSIS | 0.07106 | 2.158285 | 0.031743 |
| HALLMARK_ESTROGEN_RESPONSE_LATE | 0.043278 | 1.769359 | 0.077906 |
| HALLMARK_PROTEIN_SECRETION | 0.068088 | 1.759043 | 0.079646 |
| HALLMARK_DNA_REPAIR | 0.058867 | 1.591479 | 0.112613 |
| HALLMARK_IL2_STAT5_SIGNALING | 0.037703 | 1.577492 | 0.115794 |
| HALLMARK_BILE_ACID_METABOLISM | 0.035362 | 1.513027 | 0.131383 |
| HALLMARK_IL6_JAK_STAT3_SIGNALING | 0.057035 | 1.45824 | 0.145878 |
| HALLMARK_UV_RESPONSE_DN | 0.04069 | 1.440672 | 0.150778 |
| HALLMARK_MTORC1_SIGNALING | 0.064074 | 1.438855 | 0.151291 |
| HALLMARK_MYC_TARGETS_V2 | 0.075293 | 1.324724 | 0.186326 |
| HALLMARK_ANDROGEN_RESPONSE | 0.032391 | 1.216385 | 0.224847 |
| HALLMARK_CHOLESTEROL_HOMEOSTASIS | 0.03408 | 0.987437 | 0.324268 |
| HALLMARK_WNT_BETA_CATENIN_SIGNALING | 0.02952 | 0.825423 | 0.409823 |
| HALLMARK_ADIPOGENESIS | 0.022667 | 0.792966 | 0.428459 |
| HALLMARK_MITOTIC_SPINDLE | 0.019152 | 0.75139 | 0.453039 |
| HALLMARK_APOPTOSIS | 0.023873 | 0.722336 | 0.470682 |
| HALLMARK_FATTY_ACID_METABOLISM | 0.019513 | 0.654705 | 0.513187 |
| HALLMARK_G2M_CHECKPOINT | 0.023926 | 0.614241 | 0.539547 |
| HALLMARK_E2F_TARGETS | 0.032349 | 0.608081 | 0.543619 |
| HALLMARK_ALLOGRAFT_REJECTION | 0.012423 | 0.453804 | 0.650316 |
| HALLMARK_INTERFERON_ALPHA_RESPONSE | 0.014814 | 0.350571 | 0.72617 |
| HALLMARK_UNFOLDED_PROTEIN_RESPONSE | 0.012127 | 0.293527 | 0.769334 |
| HALLMARK_XENOBIOTIC_METABOLISM | 0.005411 | 0.243205 | 0.808022 |
| HALLMARK_P53_PATHWAY | 1.00E-05 | 0.000426 | 0.99966 |
| HALLMARK_ESTROGEN_RESPONSE_EARLY | -0.00308 | -0.13364 | 0.893786 |
| HALLMARK_REACTIVE_OXIGEN_SPECIES_PATHWAY | -0.01786 | -0.40508 | 0.68572 |
| HALLMARK_INTERFERON_GAMMA_RESPONSE | -0.01548 | -0.40544 | 0.685462 |
| HALLMARK_SPERMATOGENESIS | -0.01067 | -0.52375 | 0.600858 |
| HALLMARK_PANCREAS_BETA_CELLS | -0.02487 | -0.76279 | 0.446224 |
| HALLMARK_KRAS_SIGNALING_UP | -0.01996 | -0.83779 | 0.40285 |
| HALLMARK_PEROXISOME | -0.0562 | -1.68246 | 0.093577 |
| HALLMARK_KRAS_SIGNALING_DN | -0.04015 | -1.77042 | 0.077729 |
| HALLMARK_ANGIOGENESIS | -0.09192 | -1.79673 | 0.07344 |
| HALLMARK_UV_RESPONSE_UP | -0.05585 | -1.81837 | 0.07006 |
| HALLMARK_EPITHELIAL_MESENCHYMAL_TRANSITION | -0.04981 | -1.96824 | 0.050012 |
| HALLMARK_HEDGEHOG_SIGNALING | -0.08068 | -2.05235 | 0.041051 |
| HALLMARK_COMPLEMENT | -0.07347 | -2.29665 | 0.022365 |
| HALLMARK_APICAL_JUNCTION | -0.05219 | -2.36058 | 0.018921 |
| HALLMARK_MYOGENESIS | -0.0593 | -2.36281 | 0.01881 |
| HALLMARK_COAGULATION | -0.06946 | -2.4298 | 0.015726 |
| HALLMARK_HEME_METABOLISM | -0.13777 | -2.52146 | 0.012234 |
| HALLMARK_TNFA_SIGNALING_VIA_NFKB | -0.11265 | -2.52866 | 0.011991 |
| HALLMARK_HYPOXIA | -0.06572 | -2.63283 | 0.008931 |
| HALLMARK_INFLAMMATORY_RESPONSE | -0.08763 | -2.79325 | 0.005572 |
| HALLMARK_APICAL_SURFACE | -0.0904 | -2.8216 | 0.005115 |
