# Supplementary material for: Dysregulation of miR-138-5p/RPS6KA1-AP2M1 Is Associated With Poor Prognosis in AML
Source: Front Cell Dev Biol. 2021 Feb 26;9:641629. doi: 10.3389/fcell.2021.641629 (PMC7959750; doi:10.3389/fcell.2021.641629)
Supplement: Supplementary Figure 1 — Clustering dendrograms of genes based on a dissimilarity measure (1-TOM). [file Data_Sheet_1.ZIP › supplemental materials/Table S6.docx]

**Table S6: The enriched terms of BP for the genes in green module.**

| Term | Count | Ratio (%) | P-Value |
| --- | --- | --- | --- |
| GO:0018105~peptidyl-serine phosphorylation | 7 | 0.06525 | 5.82E-06 |
| GO:0000082~G1/S transition of mitotic cell cycle | 5 | 0.046607 | 2.49E-05 |
| GO:0008284~positive regulation of cell proliferation | 5 | 0.046607 | 0.014635 |
| GO:0018107~peptidyl-threonine phosphorylation | 4 | 0.037286 | 0.000281 |
| GO:0007050~cell cycle arrest | 4 | 0.037286 | 0.001204 |
| GO:0043066~negative regulation of apoptotic process | 4 | 0.037286 | 0.057991 |
| GO:0008285~negative regulation of cell proliferation | 4 | 0.037286 | 0.062857 |
| GO:0090398~cellular senescence | 3 | 0.027964 | 0.001598 |
| GO:0046777~protein autophosphorylation | 3 | 0.027964 | 0.081139 |
| GO:0008283~cell proliferation | 3 | 0.027964 | 0.09409 |
| GO:0008543~fibroblast growth factor receptor signaling pathway | 3 | 0.027964 | 0.008292 |
| GO:0045429~positive regulation of nitric oxide biosynthetic process | 3 | 0.027964 | 0.002658 |
| GO:0045740~positive regulation of DNA replication | 3 | 0.027964 | 0.003969 |
| GO:0048146~positive regulation of fibroblast proliferation | 3 | 0.027964 | 0.008803 |
| GO:0032436~positive regulation of proteasomal ubiquitin-dependent protein catabolic process | 3 | 0.027964 | 0.020988 |
| GO:0010629~negative regulation of gene expression | 3 | 0.027964 | 0.032684 |
| GO:0007265~Ras protein signal transduction | 3 | 0.027964 | 0.007312 |
| GO:0008286~insulin receptor signaling pathway | 3 | 0.027964 | 0.012757 |
| GO:0033206~meiotic cytokinesis | 2 | 0.018643 | 0.008585 |
| GO:0051653~spindle localization | 2 | 0.018643 | 0.021327 |
| GO:0034314~Arp2/3 complex-mediated actin nucleation | 2 | 0.018643 | 0.054526 |
| GO:0071850~mitotic cell cycle arrest | 2 | 0.018643 | 0.029733 |
| GO:0008356~asymmetric cell division | 2 | 0.018643 | 0.008585 |
| GO:0006611~protein export from nucleus | 2 | 0.018643 | 0.078697 |
| GO:0007163~establishment or maintenance of cell polarity | 2 | 0.018643 | 0.062651 |
| GO:0016344~meiotic chromosome movement towards spindle pole | 2 | 0.018643 | 0.008585 |
| GO:0031659~positive regulation of cyclin-dependent protein serine/threonine kinase activity involved in G1/S transition of mitotic cell cycle | 2 | 0.018643 | 0.025539 |
| GO:0031954~positive regulation of protein autophosphorylation | 2 | 0.018643 | 0.054526 |
| GO:1900182~positive regulation of protein localization to nucleus | 2 | 0.018643 | 0.054526 |
| GO:0045736~negative regulation of cyclin-dependent protein serine/threonine kinase activity | 2 | 0.018643 | 0.070708 |
| GO:2000379~positive regulation of reactive oxygen species metabolic process | 2 | 0.018643 | 0.070708 |
| GO:0010800~positive regulation of peptidyl-threonine phosphorylation | 2 | 0.018643 | 0.074711 |
| GO:0045931~positive regulation of mitotic cell cycle | 2 | 0.018643 | 0.082667 |
| GO:0032007~negative regulation of TOR signaling | 2 | 0.018643 | 0.090555 |
| GO:0005977~glycogen metabolic process | 2 | 0.018643 | 0.074711 |
| GO:0006983~ER overload response | 2 | 0.018643 | 0.029733 |
| GO:0071380~cellular response to prostaglandin E stimulus | 2 | 0.018643 | 0.050437 |
| GO:0006813~potassium ion transport | 2 | 0.018643 | 0.058597 |
| GO:0070141~response to UV-A | 2 | 0.018643 | 0.017098 |

Note. BP, biological process.
