# Supplementary material for: Dysregulation of miR-138-5p/RPS6KA1-AP2M1 Is Associated With Poor Prognosis in AML
Source: Front Cell Dev Biol. 2021 Feb 26;9:641629. doi: 10.3389/fcell.2021.641629 (PMC7959750; doi:10.3389/fcell.2021.641629)
Supplement: Supplementary Figure 1 — Clustering dendrograms of genes based on a dissimilarity measure (1-TOM). [file Data_Sheet_1.ZIP › supplemental materials/Table S7.docx]

**Table S7: The enriched terms of CC for the genes in green module.**

| Term | Count | Ratio (%) | P-Value |
| --- | --- | --- | --- |
| GO:0070062~extracellular exosome | 20 | 0.186428 | 0.000438 |
| GO:0005829~cytosol | 10 | 0.093214 | 0.0105 |
| GO:0005730~nucleolus | 7 | 0.06525 | 0.022575 |
| GO:0005769~early endosome | 4 | 0.037286 | 0.014857 |
| GO:0000307~cyclin-dependent protein kinase holoenzyme complex | 3 | 0.027964 | 0.002154 |
| GO:0005911~cell-cell junction | 3 | 0.027964 | 0.064317 |
| GO:0005768~endosome | 3 | 0.027964 | 0.099175 |
| GO:0005885~Arp2/3 protein complex | 2 | 0.018643 | 0.032303 |

Note. CC, cellular component.
