# Supplementary material for: Dysregulation of miR-138-5p/RPS6KA1-AP2M1 Is Associated With Poor Prognosis in AML
Source: Front Cell Dev Biol. 2021 Feb 26;9:641629. doi: 10.3389/fcell.2021.641629 (PMC7959750; doi:10.3389/fcell.2021.641629)
Supplement: Supplementary Figure 1 — Clustering dendrograms of genes based on a dissimilarity measure (1-TOM). [file Data_Sheet_1.ZIP › supplemental materials/Table S8.docx]

**Table S8: The enriched terms of MF for the genes in green module.**

| Term | Count | Ratio (%) | P-Value |
| --- | --- | --- | --- |
| GO:0005524~ATP binding | 14 | 0.1305 | 5.62E-04 |
| GO:0004674~protein serine/threonine kinase activity | 5 | 0.046607 | 0.010161 |
| GO:0004693~cyclin-dependent protein serine/threonine kinase activity | 3 | 0.027964 | 4.61E-04 |
| GO:0030235~nitric-oxide synthase regulator activity | 2 | 0.018643 | 0.008246 |
| GO:0035173~histone kinase activity | 2 | 0.018643 | 0.012344 |
| GO:0004861~cyclin-dependent protein serine/threonine kinase inhibitor activity | 2 | 0.018643 | 0.032588 |
| GO:0004712~protein serine/threonine/tyrosine kinase activity | 2 | 0.018643 | 0.060247 |
| GO:0043325~phosphatidylinositol-3,4-bisphosphate binding | 2 | 0.018643 | 0.060247 |
| GO:0005547~phosphatidylinositol-3,4,5-trisphosphate binding | 2 | 0.018643 | 0.098422 |

Note. MF, molecular function.
